# Biomaterials Adapted to Vat Photopolymerization in 3D Printing: Characteristics and Medical Applications

**DOI:** 10.3390/jfb15010007

**Published:** 2023-12-22

**Authors:** Iosif-Aliodor Timofticiuc, Octavian Călinescu, Adrian Iftime, Serban Dragosloveanu, Ana Caruntu, Andreea-Elena Scheau, Ioana Anca Badarau, Andreea Cristiana Didilescu, Constantin Caruntu, Cristian Scheau

**Affiliations:** 1 Department of Physiology, The “Carol Davila” University of Medicine and Pharmacy, 8 Eroii Sanitari Boulevard, 050474 Bucharest, Romania; 2 Department of Biophysics, The “Carol Davila” University of Medicine and Pharmacy, 8 Eroii Sanitari Boulevard, 050474 Bucharest, Romania; 3 Department of Orthopaedics and Traumatology, The “Carol Davila” University of Medicine and Pharmacy, 050474 Bucharest, Romania; 4 Department of Orthopaedics, “Foisor” Clinical Hospital of Orthopaedics, Traumatology and Osteoarticular TB, 021382 Bucharest, Romania; 5 Department of Oral and Maxillofacial Surgery, “Carol Davila” Central Military Emergency Hospital, 010825 Bucharest, Romania; 6 Department of Oral and Maxillofacial Surgery, Faculty of Dental Medicine, Titu Maiorescu University, 031593 Bucharest, Romania; 7 Department of Radiology and Medical Imaging, Fundeni Clinical Institute, 022328 Bucharest, Romania; 8 Department of Embryology, Faculty of Dentistry, The “Carol Davila” University of Medicine and Pharmacy, 8 Eroii Sanitari Boulevard, 050474 Bucharest, Romania; 9 Department of Dermatology, “Prof. N.C. Paulescu” National Institute of Diabetes, Nutrition and Metabolic Diseases, 011233 Bucharest, Romania; 10 Department of Radiology and Medical Imaging, “Foisor” Clinical Hospital of Orthopaedics, Traumatology and Osteoarticular TB, 021382 Bucharest, Romania

**Keywords:** 3D printing, digital light processing, stereolitography, vat photopolymerization, biocompatible, additive manufacturing

## Abstract

Along with the rapid and extensive advancements in the 3D printing field, a diverse range of uses for 3D printing have appeared in the spectrum of medical applications. Vat photopolymerization (VPP) stands out as one of the most extensively researched methods of 3D printing, with its main advantages being a high printing speed and the ability to produce high-resolution structures. A major challenge in using VPP 3D-printed materials in medicine is the general incompatibility of standard VPP resin mixtures with the requirements of biocompatibility and biofunctionality. Instead of developing completely new materials, an alternate approach to solving this problem involves adapting existing biomaterials. These materials are incompatible with VPP 3D printing in their pure form but can be adapted to the VPP chemistry and general process through the use of innovative mixtures and the addition of specific pre- and post-printing steps. This review’s primary objective is to highlight biofunctional and biocompatible materials that have been adapted to VPP. We present and compare the suitability of these adapted materials to different medical applications and propose other biomaterials that could be further adapted to the VPP 3D printing process in order to fulfill patient-specific medical requirements.

## 1. Introduction

Additive manufacturing, commonly known as 3D printing, has undergone a tremendous evolution over the last few decades, now having applications in nearly all fields of industry, academia, and medicine [1]. Unlike traditional, subtractive manufacturing techniques, which remove material in order to manufacture an object, 3D printing techniques build an object layer by layer, following a geometry defined by a computer model designed by specialized computer-aided design (CAD) software [1,2,3,4].

In the medical field, 3D printing currently has a wide variety of uses, including the building of custom models of a patient’s anatomy as an aid in surgery, providing models for training, patient-specific reconstructed models for surgical planning, the manufacture of custom prostheses, and the reproduction of tissues and organs for research [1,5]. With the recent push towards personalized medicine, 3D printing can also be employed to manufacture pharmaceutical formulations with composition, dosage, and release profiles tailor-made for each individual patient [6].

A multitude of 3D printing technologies have been developed, each with its own dedicated materials adapted to a particular mode of layer deposition. The current classification, according to ISO/ASTM52900-21 (Additive manufacturing—General principles—Fundamentals and vocabulary) [7], lists seven categories in which 3D printing technologies can be classified: material extrusion, material jetting, binder jetting, powder bed fusion, directed energy deposition, sheet lamination, and vat photopolymerization (VPP) [4,8,9].

The development of 3D printing technology has paved the way for two important directions for applications in the healthcare industry. On the one hand, a constant need for prostheses or substituents of different parts of the body in the fields of dentistry, orthopedics, and general or oral and maxillofacial surgery has resulted in the building of cheaper, faster, more precise, personalized replacement constructs that significantly improve patient outcomes and quality of life [10,11,12]. On the other hand, 3D printing brings advantages to tissue engineering, an emerging technology aimed at overcoming limitations in organ and tissue transplantation [13,14]. Bone scaffolds for tissue engineering have been developed using various methods, such as the Fused Deposition Material technique—which can facilitate optimal mechanical properties—or the use of magnetic scaffolds, therefore allowing for the placement and orientation of cells in a biological setting [15,16,17]. The development of a bone scaffold using polycaprolactone (PCL) as the scaffold and chitosan hydrogel as the bioactive component presented optimal mechanical properties (a compressive strength of 6.7 MPa, close to the natural cancellous bone compressive strength) and optimal pores with a size of around 350 µm that were filled with hydrogel [17]. Moreover, a tracheal model was proposed as a solution for organ replacement, and one of the greatest advantages was the partial (10–20%) tissue deformity for forces of 20N that allowed for an effective biological function [18]. The nervous system was also addressed during the development of sustainable solutions using 3D printing, and a method for peripheral nerve regeneration was introduced by building a 3D-printed model from cryo-polymerized GelMA gel [19].

One of the current limitations of 3D-printed materials is that they lack specific bioactive properties that will allow for their integration and function at the implant site. However, new methods have arisen that enable the incorporation of desired substances with required characteristics. After designing a scaffold with various mechanical properties using conventional 3D printing methods, the inclusion of biomaterials with biological properties is possible through the development of VPP. In theory, using the right mixtures, any type of material can be transformed into a resin-like slurry printable by VPP; therefore, this technique has emerged as one of the most used in the medical field, with the greatest potential for building structures with specific biological functions [20,21].

**Figure 1:**
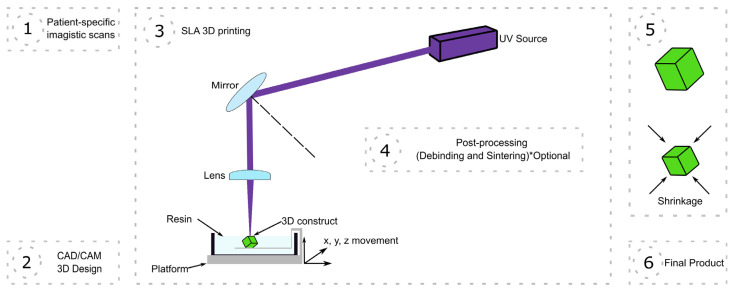
Stereolithography (SLA) 3D printing from patient-specific analysis to final product. CAD = computer-aided design; CAM = computer-aided manufacturing. * various post-processing options: most of the time, debinding and sintering are necessary, but not mandatory; moreover, other post-processing methods such as heat treatment or alcohol baths can be applied.

**Figure 2:**
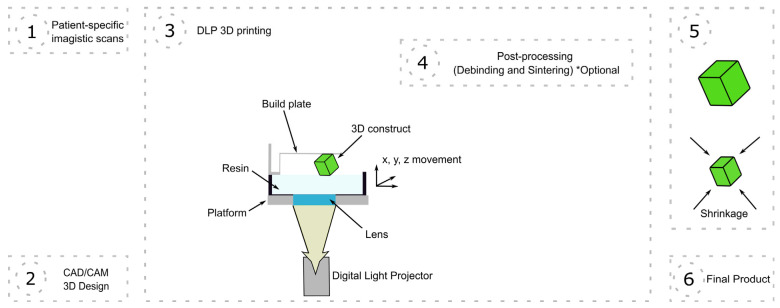
Digital light projector (DLP) 3D printing from patient-specific analysis to final product. CAD = computer-aided design; CAM = computer-aided manufacturing. * various post-processing options: most of the time, debinding and sintering are necessary, but not mandatory; moreover, other post-processing methods such as heat treatment or alcohol baths can be applied.

VPP was the first proposed 3D printing technique [22], and it is of particular interest in the biomedical field as it can rapidly produce complex structures with high resolution [23]. In brief, VPP uses liquid resin monomers or oligomers that are polymerized when exposed to a light source of a specific wavelength [24]. Several variations of VPP exist, with, historically speaking, the first one developed being stereolithography (SLA) (Figure 1), which uses a UV laser beam that travels across a programmed path, curing the resin that it contacts [25]. Consequently, as the laser needs to eventually move and trace the entire volume of the object being printed pixel by pixel, SLA is relatively slow. Mask projection VPP techniques such as those that employ a digital light projector (DLP) (Figure 2) or a liquid crystal display (LCD) instead expose an entire layer of resin to light at a time, masking the areas which do not need to be cured [26]. The continuous light interface production (CLIP) technique uses a more advanced projector and a polymerization-inhibiting oxygen layer above the base of the vat, and it allows for continuous printing at speeds up to a hundred times higher than SLA or DLP [23]. Finally, if a sub-micrometer resolution is required, two-photon polymerization (TPP) can be used, which involves the use of two femtosecond laser beams, although the sizes of the printed objects that can be derived from this technique are currently limited [27].

A major challenge in employing VPP for the 3D printing of materials to be used in the human body is biocompatibility [28]. For a 3D-printed material to be biocompatible, it has to fulfil several conditions besides being printable, including having appropriate mechanical properties, having safe degradation byproducts, good degradation kinetics, and exhibiting biomimicry [29]. Typical photosensitive resins contain acrylates or methacrylates as the monomer, as well as photoinitiators and photostabilizers that can be toxic to cells [30]. Consequently, there is a high interest in developing novel biocompatible materials for VPP 3D printing. Rather than developing completely new material chemistries for this purpose, an alternate approach is adapting materials that have already been shown to be biocompatible to the VPP 3D printing process.

Several reviews on the practical applications of VPP in medicine are available, presenting the technological aspects, postprocessing methods, or properties of certain materials used in a specific field, such as orthopedics or dentistry [21,31,32,33]. However, we could not identify a comprehensive review of all of the specific properties and functionalities of the biomaterials adapted for VPP that considered the entire spectrum of their potential applications.

Therefore, this paper aims to report on the developments in adapting existing biomaterials that are generally incompatible with VPP in their pure form to be used as the basis for novel biomaterials adapted for vat photopolymerization. We will review the current state of the art and list the main types of materials that have been adapted to the VPP 3D printing process so far while also listing the medical applications of the respective materials. The data presented here may serve as a starting point and a guide for research teams aiming to include 3D printing in their practice, containing essential reported information on the materials used in VPP.

## 2. The Considerations and Challenges of Adapting Biomaterials to VPP

**Figure 3:**
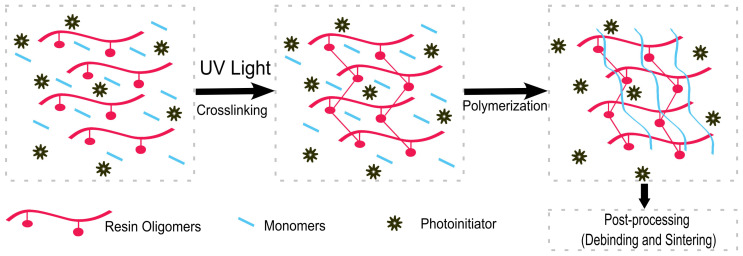
The processes of photocrosslinking and polymerisation that occur before 3D printing and during 3D printing.

Vat photopolymerization employs a suitable liquid mixture of monomers and oligomers that can be polymerized upon exposure to light-forming thermosets [24]. Usually, ultraviolet (UV) radiation is used for the photopolymerization process, although there is a current push to use longer wavelengths of light (visible up to near infrared) in order to overcome some limitations of UV radiation [24]. The resin mixture requires the presence of a photoinitiator that can efficiently absorb light and produces, in response, reactive species that promote the growth of polymer chains [24] (Figure 3). The process of photopolymerization is irreversible, as polymers cannot be returned to their monomeric, liquid form [22]. In addition to the monomers and photoinitiators, other additives can be employed in order to modulate the properties of the final material, as well as the behavior of the liquid resin during the printing process [24]. For example, a photoabsorber is often used in order to improve the resolution of the final print by reducing the penetration depth of the light in the uncured resin [34].

Employing 3D-printed materials using VPP in a biomedical context presents a particular set of challenges regarding both the toxicity and the desired properties of the final material. The most utilized resins in VPP contain (meth)acrylate monomers that are polymerized by a radical system following the cleavage of the photoinitiator by light [24,35]. While the polymerized material is not necessarily toxic, the unreacted monomers as well as the initiator generally are [36]. These have to be removed via extensive washing using a solvent such as ethanol or isopropyl alcohol [37]. However, this procedure can negatively affect the mechanical properties of the 3D-printed material [37]. Following washing, an additional post-curing step wherein the 3D-printed object is again exposed to UV light in a post-curing chamber is required in order to ensure complete polymerization and cross-chain formation [38]. This step will also affect the final mechanical properties of the resin, and shrinking of the final print is generally observed [39].

Even when using extensive post-treatment procedures, caution must still be taken that the final product is non-toxic. A recent study [40] has shown that, even when all the recommended post-print procedures are followed, cytotoxicity might still be present even for resins that are certified to be biocompatible. Thus, two dental resins printed using SLA and certified for use in the manufacture of oral surgical guides and retainers were shown to be toxic in vitro to mammalian oocytes [40]. In the case of one of the resins, the responsible compound was identified to be a light stabilizer, Tinuvin 292, that leached from the printed product [40].

The use of additives introduces additional constraints to the 3D printing process. For instance, one of the most desired effects that we will also describe further in this review is the VPP 3D printing of ceramic materials. In order to achieve this, ceramic particles are added to the liquid resin mixture. In this case, both the size of the ceramic particles and the overall effect on the viscosity of the resin have to be considered [36,41]. The resulting 3D-printed object will consist of a polymer–ceramic composite material which is generally stronger than a pure polymer [36]. If a purely ceramic object is desired, the resulting 3D-printed object in its “green”, unprocessed form can be further treated. The organic polymer is removed through thermal debinding (pyrolysis) [36]. Finally, sintering is used to strengthen the remaining ceramic [41,42]. Depending on the composition of the material, sintering time, and temperature, different degrees of shrinkage can occur in the final product, and this has to be taken into account in the design of the CAD 3D model [43,44].

## 3. Biomaterials Adapted to VPP 3D Printing

Adapting biomaterials for VPP 3D printing comes as a response to the patients’ needs for specific, rapid, and precise constructions that yield the appropriate biological characteristics. In the following section, we summarize the latest and most pertinent studies showcasing the full potential of these biomaterials in both 3D printing and the medical field. Each biomaterial has certain limitations and specific applications in the healthcare industry, as well as the potential for future integration in the domains of 3D printing and medicine.

Biomaterials may be of organic or inorganic nature, and when considering the process, there are no significant differences reported in the 3D printing between these categories, as each material shows limitations and specific methods for adaptation to VPP. However, a relevant difference lies in the temporal and procedural requisites for reaching their final (synthetic) form. Organic biomaterials, in particular, require additional chemical steps, the most relevant involving the need for the polymerization of an organic compound (to be distinguished from the polymerization occurring during the 3D printing process) under specific reaction conditions.

### 3.1. Inorganic Biomaterials

#### 3.1.1. Hydroxyapatite (Hap)

Hydroxyapatite, the chemical formula of which is Ca_10_(OH)_2_(PO4)_6_, is an inorganic compound that is a key constituent of the mineralized portion of bones [45]. It is one of the most used and relevant ceramic biomaterials in the realm of 3D printing [46,47]. Although research reporting the use of 3D-printed hydroxyapatite using VPP is limited, certain studies have illustrated that the resulting product exhibits increased precision and enhanced sensitivity and accuracy compared to alternative 3D printing technologies [48]. Hap 3D-printed constructs have excellent bioactive properties, including increased biocompatibility, osteoinductivity, osteoconductivity, excellent bioresorbability and biodegradation, and near-to-zero cytotoxicity [49,50,51,52,53]. These qualities hold significant importance in medical applications such as tissue engineering for bone grafts [49,50,51,53], dental root implants [52], and coatings for 3D-printed metallic implants [54,55,56].

Good results have been obtained by employing Hap in the 3D printing of bone grafts for bone regeneration. It is known that the pore sizes and shapes of these builds are the main modulators for enhanced or diminished osteoconductive properties [51,53]. Research has demonstrated that larger pore sizes (exceeding 300 μm) enhance the osteogenesis process [57,58], although the challenge lies in achieving a balance between pore size and shape (with cubic pores being more efficient [50]) while maintaining the required mechanical performance [59].

Printed with the same technology are Hap dental roots. In oral surgery, 3D printing parts of teeth has gained popularity. The most used and studied biocompatible material in this particular field is currently zirconium [33], but recent studies have shown some promising results using DLP 3D-printed hydroxyapatite [52]. There are still challenges in finding the ideal alloys that can also enhance the bacteriostatic properties [52].

Despite not being printed via VPP, hydroxyapatite coatings are representative of the compound’s bioactive properties [54]. As most orthopedic implants are composed of biocompatible metals such as titanium due to their superior biomechanical properties [60], there are still some challenges with the biointegration of the implants, which can be effectively addressed through Hap coatings or Hap-ion coatings [55].

Hydroxyapatite is a material with untapped potential. Some studies propose its usage in building feasible, sophisticated, and complex scaffolds that maintain the requirements of an artificial bone scaffold, or even bioactive and biocompatible mixtures with extracellular matrix proteins that can promote cellular integration and responsiveness [53]. Possible enhancements in strength have also been reported, but the architecture and the porosities of the builds require further study [51]. Furthermore, the development of antimicrobial implants in the fields of orthopedics and neurosurgery is also currently being explored [55].

#### 3.1.2. Zirconia

Zirconia, a crystalline oxide of zirconium (ZrO_2_), can be shaped into a monolithic form suitable for medical applications. It stands out in the realm of dental ceramics due to its exceptional mechanical properties, rendering it both the strongest and most aesthetically pleasing ceramic [33,61,62]. DLP is one of the most advanced 3D printing techniques used in the fabrication of ceramics due to its high speed and increased precision and accuracy [63], as well as the fact that zirconia can be employed as a material [33].

Despite not being inherently bioactive, zirconia-based structures find extensive use in dentistry [64] thanks to a multitude of crucial properties, including rheological behavior, curing characteristics, biocompatibility, and mechanical and tribological performance [33,65,66]. Factors such as resistance to bacterial colonization, low toxicity, and enhanced fracture strength are essential for the use of zirconia in dental implants [64,67,68]. Unalloyed zirconia is suitable for dental crowns, bridges, implants, and abutments, as well as for teeth repair, as it can resist compressive strengths as high as 2000 Mpa [33,69]. Despite other materials being used in the past for dental implants, zirconia or other dental resin-based materials have gained popularity in the field due to rises in patient quality of life because of their clinical performance and aesthetic quality [66]. Zirconia does not cause adverse reactions when interacting with oral tissues. Additional advantages of dental applications include chemical stability over time and the capacity for rapid printing [10].

Zirconia stabilized with yttrium oxide (yttria) is an outstanding ceramic material used in dentistry due to its high stability and enhanced fracture strength [70]. Comparative studies involving zirconia stabilized with 3 mol% and 8 mol% of yttria have demonstrated improved stability and printability when compared to unstabilized zirconia. There were minimal differences in printability observed between these two yttria concentrations when employing DLP printing technology [65]. Research showed that the addition of yttria-stabilized zirconia in 3D dental constructs leads to enhanced thermomechanical and biological properties [66].

As previously mentioned, zirconia lacks bioactive properties. Consequently, it finds applications not only in dentistry but also in orthopedic implants [71]. To achieve the desired osteoinductivity and osteoconductivity properties of the orthopedic implants, zirconia-based builds must be coated with bioactive materials [72]. One extensively studied coating material is hydroxyapatite [73]. Such combinations are frequently utilized due to the outstanding mechanical properties of zirconia and the great bioactivity of hydroxyapatite [67].

Despite its suitability for applications demanding high mechanical and fracture strength, zirconia does come with certain drawbacks when adapting it for VPP. Notably, its high refractive index (2.1) [74], while advantageous for dental implants in terms of aesthetics, poses challenges in DLP technology reliant on UV light [33]. Furthermore, zirconia’s fragility can lead to crack formation in the build, unless it is combined with various slurries [64]. Also to be considered are the different orientations of the prints, with studies showing that orientating the 3D model at 45 degrees exhibited the greatest resistance to indentation fracture [75].

Zirconia was adapted for DLP 3D printing due to the technique’s increased speed and accuracy in fabricating patient-specific dental parts. Data in the literature allude to the possibility of adapting zirconia for suitability with DLP printers by dispersing zirconia particles using silane as a coupling agent. However, further investigations are required to assess its performance under different physical conditions and mechanical stress [64].

#### 3.1.3. Lithium Disilicate

Lithium disilicate (Li_2_Si_2_O_5_) is categorized as a glass–ceramic material with a biphasic polycrystalline structure [76]. Owing to its remarkable mechanical properties and aesthetic qualities, lithium disilicate has gained prominence in the field of dental restorations [77]. Manipulating the material with traditional techniques such as conventional restoration waxing exhibited drawbacks related to precision, accuracy, and susceptibility to human error [78]. However, due to the high precision and speed offered by DLP 3D printing, along with advancements in 3D printing techniques [63], such as LCM-Lithography-based Ceramics (DLP in conjunction with highly filled ceramic suspensions of photocurable resins), 3D-printed dental components made from lithium disilicate have shown promise [79].

Although limited research has been conducted on 3D-printed lithium disilicate, it was reported that lithium disilicate is suitable in dental restorations, as it meets both the aesthetic and functional requirements for the anterior and posterior regions of the oral cavity [78]. Dental crowns and veneers are the primary structures widely employed in this context [79,80,81,82]. A recent study showed that lithium disilicate builds overcome the limitations of metal–ceramic restorations, which have long been considered the gold standard in dental restorations [79,81].

In veneers, studies comparing 3D-printed parts with the manual waxing technique reported that the 3D-printed build displayed no differences in the quality of the final product regarding the marginal and internal fit. Instead, it came only with advantages, such as the ability to rigorously replicate the dental part and the accuracy and the speeds of the builds [82,83]. Crucial to veneer fabrication are the mechanical properties of the material used (in this case, with reported values for fracture toughness of 3.3 ± 0.3 Mpa m^1/2^ [84] and flexural strength of 280 Mpa) [85]. Additionally, LCM lithium disilicate veneers have shown satisfactory aesthetics and an acceptable marginal fit [79].

Several studies have demonstrated that lithium disilicate glass ceramics stand out as an excellent option for dental rehabilitation, particularly for producing monolithic crowns [86]. Similar to veneers, 3D printing a crown is an efficient and controlled process, with fewer errors and less materials being wasted compared with traditional waxing. It was also reported that VPP techniques are more efficient than other recent methods of producing crowns and veneers, such as the subtractive CAD/CAM technique [78]. A few analyses revealed that the lithium disilicate dental parts are adequate for patients requiring restorations with a lifespan exceeding 5 to 9 years, considering that the complication rate is usually under 20% [80,81].

Alongside the previously mentioned advantages, we also need to mention the challenges that occur during 3D printing and post-processing. One notable drawback is the susceptibility of builds, particularly crowns, to fracture, with posterior and molar crowns being more prone to this issue [80]. Furthermore, the incidence of fracture presumably increases after a certain time of clinical service and also increases with the forces developed in the mastication process [80]. Another concern arises from post-processing methods that can prolong the average manufacturing time and also make it less attractive for clinical practice; however, studies show that this limitation can be overcome by using the adapted DLP technique known as LCM [79]. Although 3D-printed lithium disilicate has not yet received approval for definitive restorations, there is potential for achieving this milestone in the future. Flexural and bonding strength needs to be enhanced to fully apply this material in all dental applications, and the reduction of micro-fractures is needed [79]. Furthermore, the potential for multicolor dental part printing using lithium disilicate for aesthetic purposes is also a possibility [79].

#### 3.1.4. β-Tricalcium Phosphate (β-TCP)

Tricalcium phosphate (Ca_3_(PO_4_)_2_) exists in four different forms, the β form being relevant for this review, due to its temperature stability (below 1120 °C) and printability [87]. With a research history spanning five decades and an annual publication output exceeding 200 papers, β-tricalcium phosphate (β-TCP) has emerged as one of the most potent biocompatible materials for bone grafts [88]. With the development of additive manufacturing processes, the adaptation of β-TCP for 3D printing was an inevitable step. The current focus on the use of β-TCP in 3D printing is its compatibility with VPP 3D printing technology due to all the advantages that this technology brings, especially for manufacturing porous and high-strength scaffolds [89].

Owing to its chemical composition, β-TCP exhibits a spectrum of bioactive properties, including osteoconduction (promoting osteoblast adhesion, osteoblast proliferation, and the synthesis of new extracellular matrix components [89]); gradual biodegradation (the gradual release of ions with a therapeutic effect [90]); osteoinductivity [88]; and minimal cytotoxicity [91]. Studies have revealed that pure β-TCP scaffolds may not exhibit optimal mechanical properties due to rapid degradation in vivo, which hinders the maintenance of the build integrality before the full repair of the bone defect [92].

A recent study mentioned that, despite the pure β-TCP scaffolds in the study having a compressive strength of up to 44.7 Mpa, which is higher than the values reported in he literature for similar bioceramic scaffolds, it is essential that the ceramic framework be coated or impregnated with different biocompatible materials for enhanced properties [89]. Still, further research is required in order to facilitate compatibility between this type of mix and VPP 3D printers.

The in vivo properties of a scaffold printed via DLP technology and consisting of β-TCP mixed with pyritum (a traditional Chinese medicine composed of iron disulfide, magnesium, zinc, copper, and other compounds and elements) were recently reported in [90]. When compared with a pure β-TCP scaffold, tricalcium phosphate mixed with a pyritum scaffold managed to overcome the structural drawback of this build. With enhanced mechanical strength (approximately four times higher), improved osteoinductive properties (due to the presence of the elements mentioned above), and a lower rate of biodegradability regarding the scaffolds, the TCP/pyritum build holds promise as a biomaterial for bone defect repair [90].

Other studies have investigated the potential of carrying out bone defect repairs using β-TCP scaffolds doped with different quantities of magnesium oxide, ranging from 0 to 5%. In one study, it was revealed that Mg doping improves physicochemical functions such as mechanical strength and also slows down the degradation rate of scaffolds. Also, the biological functions were improved, and the Mg-doped DLP 3D-printed scaffolds promoted the osteogenic differentiation of bone marrow stem cells and angiogenic differentiation [92]. The study also reported that the optimal doping with magnesium oxide was 3%.

#### 3.1.5. Biphasic Calcium Phosphate (BCP)

Biphasic calcium phosphate is a bioceramic that consists of a mixture of hydroxyapatite and beta-tricalcium phosphate (β-TCP) in varying ratios, with each part having a pivotal role in determining the bioactive properties of constructs fabricated using this functional biomaterial [93]. There is a scarcity of data regarding the use of BCP in VPP 3D printing with biocompatible and functional materials. Even though numerous studies concentrate on Robocasting, FDM, or SLS 3D printing technologies, there are a limited number of papers that have explored BCP builds produced through DLP 3D printing and showed that they exhibit higher precision, accuracy, and speed [94,95,96,97], although the eventual drawbacks of DLP 3D printing using BCP also need to be studied further.

**Figure 4:**
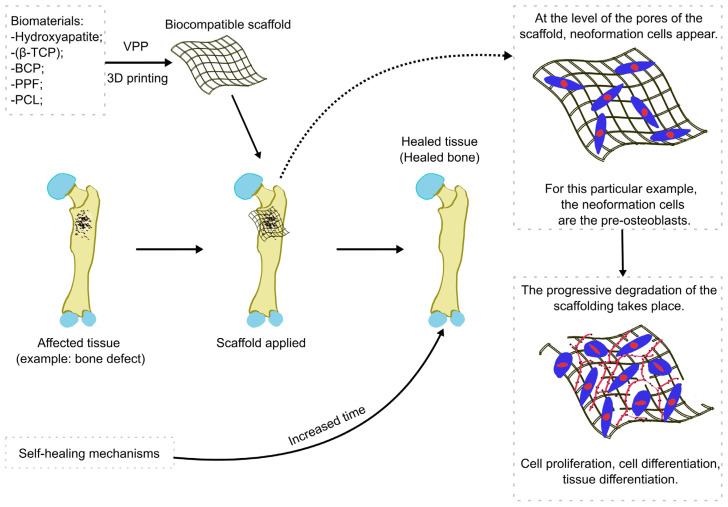
The general process of a VPP 3D-printed biocompatible scaffold applied in bone regeneration. β-TCP = beta-tricalcium phosphate, BCP = biphasic calcium phosphate, PPF = poly(propylene fumarate, PCL = polycaprolactone.

As they are composed of Hap and beta-tricalcium phosphate, BCP builds offer several bioactive properties. The osteoconductive and osteoinductive properties of Hap are crucial features that promote processes like cell migration, vascularization, and, of course, osteogenesis [98,99]. Beta-tricalcium phosphate is biodegradable, and its role in this type of construction is to provide calcium and phosphate ions for bone formation [100]. With its excellent cytocompatibility and minimal cytotoxicity, this biocompatible ceramic is well suited for manufacturing various patient-specific scaffolds in order to address bone defects [101,102] (Figure 4).

In a recent comparative study aiming to assess how pore diameter influences bone formation in the regeneration of rabbit calvarial defects, it was determined that an optimal HA/β-TCP ratio of 60:40, along with pore diameters exceeding 300 µm enhanced vascularization and new bone formation [98]. Another study comparing DLP 3D printing with 3D-milled PMMA (poly methyl methacrylate) showed that the DLP 3D-printed block displayed enhanced internal fit and demonstrated significantly greater contact with the defect surface. To elaborate, it had twice as much contact in flat defects and over 70% more contact in curved defects [103].

Additionally, the morphology of the constructs can influence their bioactive properties. For instance, analyzing composite scaffolds with a gyroid structure led to the revelation that they have excellent biocompatibility, as well as enhanced cell proliferation and adhesion [104]. The evolution of 3D additive manufacturing technology has facilitated the construction of more intricate and accurate builds, particularly in addressing patient-specific defects. Another study revealed that scaffolds incorporating oxygen-generating elements can efficiently increase oxygen levels, which is essential in preventing hypoxic cell death during the early stages of engraftment [105]. As such, biphasic calcium phosphate coated with calcium peroxide showed great potential for promoting bone ingrowth and proliferation under a hypoxic environment [105].

Despite the research on this topic being in its early stages, meaning that the disadvantages regarding DLP 3D printing with this material have yet to be revealed, biphasic calcium phosphate is not perfectly compatible with this type of technology and needs to be adapted. Similar to hydroxyapatite, mixing the bioceramics in a slurry made of acrylic monomers, dispersants, and photocatalysts is necessary; nevertheless, this might improve printing quality with DLP 3D technology, which, despite being a fast and precise method, can have drawbacks regarding the final form, the time of postprocessing, and the possible shrinkage of the build [98,102,103,104].

Further research is needed to investigate the in vivo effects of these structures and their biological behavior in order to expand their applications in the medical field [101].

### 3.2. Organic Biomaterials

#### 3.2.1. Poly(propylene fumarate)

Poly(propylene fumarate) (PPF) is a linear and unsaturated co-polyester characterized by its fumaric acid–base structure which has demonstrated a range of intriguing attributes in the medical field [106]. Due to the research carried out in the past 10 years and the development of new technologies in 3D printing, the way for faster, more precise, and increasingly complex fabrications has been paved [107]. Similar to the majority of the materials discussed in this review, slurries with various photocurable substances and minor structural adjustments are needed in order to 3D print PPF using VPP. Due to the presence of an unsaturated bond, PPF can be converted into a resin and undergo photo-crosslinking to form a VPP 3D-printable solution [108].

Another hurdle that was overcome in the goal of adapting PPF for VPP-based 3D printing is its viscosity. The intrinsic viscosity of pure PPF at 40 °C is 120 times higher than the ideal viscosity of 200 cP in 3D printing resins [109,110,111]. The lowering of the viscosity of PPF solutions was accomplished by mixing it with diethyl fumarate at various ratios, usually greater than 50% [108,111,112,113,114,115,116,117]. This ensured that the solution was printable but within the maximum limits of viscosity. Nevertheless, addressing potential defects that may arise from this approach remains an ongoing pursuit.

PPF, by itself, is not osteoinductive or osteoconductive and does not aid in bone tissue regeneration. Still, its advantageous properties, such as its great resorption, optimal degradability, low cytotoxicity, and mechanical behavior [108,113,115], in combination with the feasibility of the DLP 3D printing technique, put the PPF builds at the top of the list of functional biomaterials [116]. Studies have reported that PPF-based materials have great medical applications, including in cardiac tissue engineering, ophthalmology, and neural tissue engineering [115], yet 3D-printed PPF-based materials are more suitable in orthopedics, specifically in bone tissue engineering [113].

In one particular study wherein two DLP 3D-printed scaffolds made from PPF were used to repair murine cranial defects, it was observed that bone regeneration and material degradation depend on the molecular mass variation. It was stipulated that the PPF 1000 Da scaffold had the highest bone/interaction at 4 weeks due to the enhanced degradation of the polymer that facilitates a relative high inflammatory response [108].

In order to expand PPF scaffolds to more medical applications, these builds should be enhanced with bioactive properties [108]. However, mixing PPF with an excess of bioactive material increases the slurry viscosity and leads to printing incompatibility or defective printing. A study reported that mixing PPF with Bioglass in a range between 2.5% and 5% yielded great results in terms of surface availability and functionality, as well as cell attachment, proliferation, and osteogenic differentiation at the surface of the build [116].

Despite being studied since 1994, the compatibility of PPF with 3D printers has not been extensively investigated [112], particularly in regard to VPP. While the benefits of using PPF can be extrapolated from prior research, several challenges, including post-processing methods, the development of photoinitiators and solvents to create final, printable slurries [115], and the management of defects and cracks, necessitate further investigations. Moreover, further in vivo and in vitro studies are needed to identify the optimal concentrations to fulfill the potential of PPF tissue engineering (tissue replacement/regeneration) [115].

#### 3.2.2. Polycaprolactone (PCL) Mixes and Polycaprolactone (PCL)-Based Materials

Polycaprolactone (PCL)—(C_6_H_10_O_2_)_n_—is a synthetic polymer that can be synthesized chemically by the ring-opening polymerization of ε-caprolactone. PCL presents great biocompatibility and has been approved by the United States Food and Drug Administration (FDA) for use in humans [118,119]. A highly used material in various 3D printing technologies such as Fused Deposition Modeling (FDM) [120] and electrospinning [121], there is growing interest in adapting different biocompatible materials with distinct physico-biological attributes for VPP 3D printing techniques. A prime illustration of this venture is the integration of polycaprolactone. Although few studies have reported the use of PCL in VPP 3D printing, some authors have unveiled innovative adaptations to harness PCL’s properties in conjunction with the speed and precision of VPP [122,123,124,125].

PCL is widely favored for biomedical applications due to its remarkable characteristics, which include its low degradation rate [126], flexibility and hydrophilia [126], anisotropic behavior [127], antibacterial activity [128], adequate mechanical strength and heightened extracellular matrix gene expression [119], and biodegradability [125]. To this day, there appears to be no studies reporting the exclusive use of pure PCL in printing using VPP techniques. Some researchers have adapted and successfully implemented the properties of PCL in DLP 3D-printed structures either by incorporating a small proportion of PCL or by using PCL-based materials [122,123,124,129].

GelMA is a biopolymer employed for the 3D fabrication of different biocompatible scaffolds; however, its mechanical drawbacks do not allow for a good printing resolution using SLA or DLP. One study showed that adding PCL in a ratio of 70/30 (GelMA-PCL) increased the resolution of printing with SLA and also stabilized the viscosity [124]. To assess this novel resin blend and to challenge the 3D printer with the complexity of an anatomical structure, a human small intestine tissue scaffold was made. Furthermore, this material presented optimal cell adhesion, water swelling, softness, and precision, as well as reduced reabsorption, compared with the pure materials taken separately [124].

As mentioned, PCL-based biocompatible materials have also been tested with VPP 3D printing machines. Some studies have reported the synthesis of PCL-based urethane acrylates for DLP 3D printers [122,123]. Following synthesis, the authors of one specific study reported that the PCL-based poly(urethane acrylates) (PUA) used for 3D-printed builds had been mixed with natural resins such as polyethylene glycol diacrylate (PEGDA) or polypropylene glycol (PPG) to obtain optimal viscosity and printability. It was determined that the optimal mix with low cytotoxicity and good printability for producing tissue engineering scaffolds is the PUA-PEGDA-PPG (70:30:0) [122]. Moreover, in another study, waterborne polyurethanes produced from PCL in a ratio of 20% in the final mix were optimal for manufacturing bendable and flexible 3D architectures and devices via DLP 3D printing [123].

Another study reported on the use of PCL in the realm of 4D printing, which denotes the fourth dimension as time and considers the evolving properties of the material [129]. PCL was integrated into the scaffolds as a self-healing agent to confer this property to the system build (based on soft active materials). The study also reported drawbacks due to encountering irreparable damaged printed structures, which can lead to increased costs, but in the end, it was concluded that these types of builds have enhanced resolution, enhanced geometric complexity, and, most importantly, self-healing capabilities [129].

In the future, PCL-based materials or mixes could be applied to cartilage scaffolds [122]. Also, these resins may have great potential tissue engineering applications and, in the next decade, may become the base of artificial organs [122,123].

#### 3.2.3. Poly(methyl methacrylate)

Poly(methyl methacrylate) is a widely used synthetic polymer of significant scientific interest in the field of medical technologies, with a research history spanning over eight decades. It has been acknowledged for its favorable attributes, including its ease of processing, repairability, polishability, and good biocompatibility [130,131,132]. PMMA has found applications in various medical specialties, such as orthopedics (for bone cement and screw fixation in bone), ophthalmology (for contact and intraocular lens), neurosurgery (as filler for skull defects and for vertebrae stabilization), dental technologies, plastic surgery, and even keratoprosthesis [130,133]. With a great history regarding the methods used for manufacturing PMMA builds, from traditional ones such as pouring a fluid resin or mold-filling to more modern ones like milling blocks designed using computer-assisted design and manufacturing, the current direction is making PMMA compatible with the SLA and DLP printers [132,134].

Recent reports indicate that, for the moment, the mixes of PMMA compatible with VPP 3D printing techniques are for dental technologies and implants [135,136,137], although a few studies have reported additional medical uses [138]. The notable characteristics of PMMA-based constructs include their good aesthetics, physicochemical properties, low cost, excellent biocompatibility, low toxicity, reliability, and good mechanical strength [130,132,133].

One approach that has been found to make PMMA compatible with VPP 3D printing techniques, namely SLA 3D printing, involves blending it with other acrylates such as ethylene glycol dimethacrylate (EGDMA) and methyl methacrylate (MMA), which also present great mechanical and biological properties [134]. The study of Hata et al. aimed to find an optimal mixture for satisfactory printability and practicability following SLA 3D printing, and the authors concluded that the best mechanical, bonding, and physicochemical properties, as well as good cell viability, is given by a 30% PMMA, 56% EGDMA, 14% MMA mix [134]. This particular PMMA-based resin exhibits excellent properties, suggesting its potential suitability for various dental applications, including in crowns, bridges, denture bases, and teeth [134].

Mechanical properties are crucial in dental applications to ensure durability and resilience in patients. One study assessed the flexural strength of a DLP and SLA 3D-printed PMMA build and compared it to pieces built with a frequently used technique, namely FDM [137]. Although DLP was the fastest method for printing PMMA, all of the produced specimens fractured into several fragments when their flexural strength was tested and compared with the other two groups. Also, the specimens based on vat polymerization exhibited increased flexural strengths but suffered from numerous cracks and fractures. As the speed and precision of DLP and SLA techniques are considerable, further studies are worth pursuing in order to improve the observed disadvantages [135,136,137].

An innovative study using DLP-printed PMMA successfully built a tracheostomy tube which was later tested and functioned properly with no infections, allergies, or other reactions in rabbits [138]. It was reported that the PMMA-resin scaffold exhibited no fiber structures, contaminations, or surface cracks and also that cell viability was higher than in the control groups. The key advantages include the precision and speed of the print, the enhanced mechanical behavior pre- and post-insertion, and the absence of cytotoxic effects and visible chemical changes in vivo [138].

In summary, DLP-printed PMMA resins hold promise for advancing modern medical applications. However, they have been insufficiently studied, and not many future medical applications are in sight. Considering that compatibility with DLP and SLA 3D printers was just established, adequate in vivo testing is required, especially regarding cytotoxicity. Optimal mixes need to be identified in order to eliminate all the mechanical disadvantages that these materials currently present [134,136].

#### 3.2.4. Poly(trimethylene carbonate)

Poly(trimethylene carbonate) (PTMC) is a biocompatible and biodegradable synthetic polymer that can be manufactured through the process of ring-opening polymerization (ROP) using trimethylene carbonate (TMC), a bio-derived monomer with the following chemical formula: C_4_H_6_O_3_ [139,140,141]. PTMC is categorized as an amphiphilic biocompatible copolymer that is akin to PEG, an attention-drawing compound due to its increased compatibility with the body fluids [141]. With the development of VPP 3D printing techniques, it was concluded that PTMC is more suitable than its partner in the amphiphilic group due to superior mechanical properties and printability with DLP or SLA 3D printers [139,140].

Similarly to the previously mentioned materials adapted for VPP techniques, PTMC possesses noteworthy and distinctive properties, including flexibility, a low glass transition temperature of ≈ −20 °C, toughness, tear resistance, the absence of relevant cytotoxicity, softness, and great degradation activity suitable for building tissue engineering scaffolds [139,140,142]. As a synthetic material, it does not present bioactivity, but some studies have revealed different ways of transforming pure PTMC into a DLP- or SLA-printable resin with bioactive and mechanical properties [139,142].

It was stipulated that materials prepared from macromers with molecular weights higher than 10 kg mol^−1^ show enhanced mechanical properties such as high tensile strength, toughness, tear resistance, and suture retention strength [143]. A group of researchers managed to build a PTMC DLP 3D-printed structure that exceeded the 10 kg mol^−1^ molecular weight mark; specifically, it weighed 28.9 kg mol^−1^ [139]. This weight could open up new avenues in terms of the functionality of this material, mainly by making it more applicable to cases that require constructs to be subjected to great forces, such as the case with meniscus implants [139].

Another biofunctional application is PTMC’s role in tissue engineering. In order to create a scaffold compatible with tissue engineering applications, the materials have to meet certain conditions, including being biocompatible, biodegradable, and porous while also having sufficient mechanical properties [144]. In this case, pure PTMC is very hydrophobic, but due to its high potential in medical engineering applications, a method for adapting it to DLP printing was constructed [142]. The reported solution was a mix between PTMC—a synthetic hydrophobic biopolymer—and gelatine (from porcine skin)—a natural hydrophilic biopolymer, in which a large quantity of solvent was added; the resulting mixed resin was manageable for tissue engineering applications and printable via VPP techniques [142].

Very few studies have reported the use of PTMC in additive manufacturing, and even fewer have reported on using PTMC with DLP or SLA 3D printing techniques [139,140,142]. As mentioned, the future of medical applications requires the printing of patient-specific builds quickly and with reduced costs. For now, DLP and SLA are the fastest methods, but they are limited by their incompatibilities with some biomaterials. Further research needs to identify new processes to adapt materials that show suitable properties for different biofunctional applications to these new, precise, and fast 3D printing technologies. Drug delivery and cell/stem cell therapy are also very promising fields for future research with this material or its mixes with various bioactive and biocompatible materials. Furthermore, thermogels have been reported as candidates in different potential laboratory applications [139,140].

## 4. The Medical Applications of Biomaterials Adapted for VPP

As shown in the previous section, a significant number of biocompatible materials exhibit unique characteristics pertinent to their functionality and applicability in the medical field. Of paramount significance is the fact that the presented materials lack printability in their pure form, so adapting the materials and a novel and special approach was needed in order to take advantage of the properties of newly developing 3D printing technologies (vat polymerization). Various investigations with specific methodologies have presented unique and distinguished mixtures and affirmed their functionality and diverse medical applications while assessing their printability when using light-based 3D printing technologies.

The following table (Table 1) presents the spectrum of medical applications achievable via DLP or SLA 3D printing, along with the corresponding biocompatible materials that have been documented to date. This approach might facilitate the identification of the optimal materials, 3D printers, and essential additives for slurry formulations required to attain optimal outcomes.

As previously mentioned, dentistry, orthopedics, and general surgery and oral and maxillofacial surgery are the main fields where 3D printing is extensively studied and various applications have been developed. From our experience, the medical practice must be able to build constructs that efficiently and precisely mimic all the functional and structural properties and characteristics of the patient’s specific anatomical part, and this includes the part’s mechanical properties, tissue thickness, wettability, resistance and response to pressure, elasticity, shear resistance, and response to mechanical stress. To fulfill the optimal interval for these properties in a specifically dedicated construct, it is crucial to find a material that satisfies all requirements. The table above is an aid for medical teams and a guide for specialists who choose to use 3D printers in the treatment of their patients, with crucial information that could help lead to the efficient selection of the material and printer to be used in building pieces with specific biological, chemical, and physical requirements.

## 5. Conclusions and Future Perspectives

Among the emerging applications of 3D printing are bone grafts and scaffolds. Overall, 3D-printed bone elements alleviate the drawbacks of allografts and xenografts caused by immune response complications and present enhanced osteogenic properties [145]. In our paper, we have shown that HAp, PPF, BCP, and β-TCP are biomaterials adapted for VPP with promising applications. Also, after comparing their properties (Table 1), we can conclude that HAp is the optimal material in the majority of cases for building a bony structure due to its mechanical properties and bioactive properties [146]. BCP and β-TCP present better printability, enhanced functionality, and special bioactive properties but cannot handle the same mechanical stress [120,147]. However, PPF is at the top in terms of mechanical functionality and presents a better cytotoxic response and resorption properties but lacks bioactivity, which is critical for a long-surviving graft [116].

Regarding the applications in dentistry, although ceramic–metal alloys are considered the gold standard in building dental parts, certain biomaterials have attracted interest due to their medical properties, combined with the speed and precision of VPP. Zirconia and lithium disilicate present optimal mechanical properties for dental applications, and choosing between them depends on the 3D printing technique being applied. VPP 3D-printed zirconia presents several drawbacks due to the incompatibility between UV light (the printer source) and the surface of the material [148]. With both biomaterials presenting similar physical properties, we can conclude that, in this case, lithium disilicate is more suitable for printing dental parts by VPP 3D printing. However, the two biomaterials do not present bioactive properties. In dental applications, VPP 3D-printed PMMA is the least favorable due to the numerous cracks and fractures in the final constructs [149].

In contrast with the materials developed to replace tough, hard tissues, there is increasing interest in biomaterials that are capable of mimicking soft tissues, such as PCL and PTMC, which, in our opinion could have a larger contribution in various medical specialties. Impressive and special models have been built using PCL-based materials or mixes, such as parts of the small intestine and self-healing scaffolds, while PTMC has been applied in tissue engineering scaffolds and meniscus implants [124,150,151]. There are several adapted 3D printing techniques used for building special constructs, but none of them are as fast and precise as VPP. Therefore, extensive research in this direction is encouraged. This perspective could lead to the future possibility of realizing full tissues or full organs with the capacity to self-heal and self-integrate without presenting immunological drawbacks [152,153].

**Figure 5:**
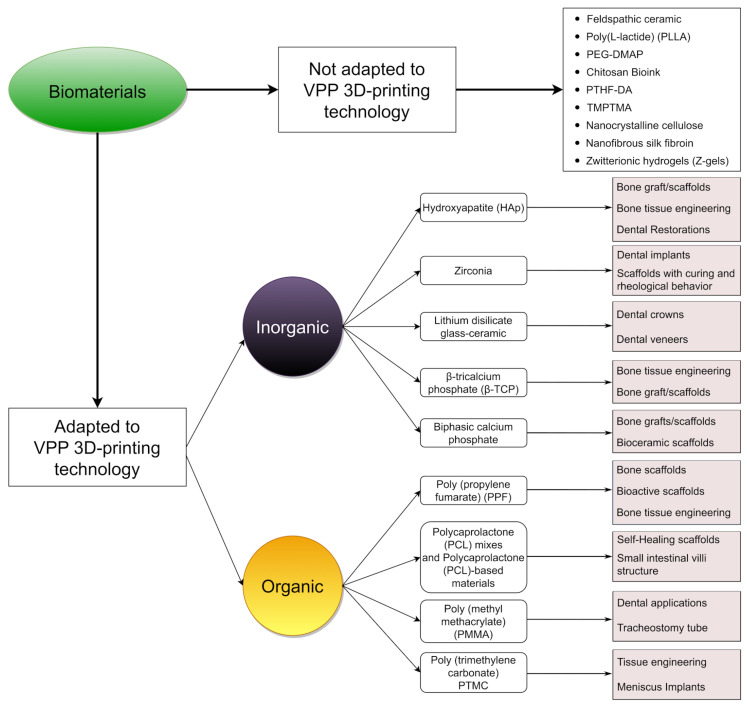
Overview of the common and emerging medical applications of biomaterials adapted for VPP 3D printing. The top row lists biomaterials that have not yet been adapted to VPP but may represent promising potential targets for adaptation.

The 3D printing of biocompatible and functional materials using VPP remains relatively unexplored. There are numerous potential avenues for future studies and the novel medical applications of the materials presented above. These materials were not originally designed for VPP 3D printing, but they were adapted and adjusted to ensure compatibility and function (Figure 5).

We must mention the other biocompatible materials reported in different studies in the last decade that have yet to be adapted for VPP techniques but have great potential due to their chemical properties and biocompatibility. Some of these materials are feldspathic ceramic [154], poly(L-lactide) (PLLA) [155], PEG-DMAP [156], chitosan Bioink [157], α,ω-polytetrahydrofuranether-diacrylate (PTHF-DA) [158], trimethylolpropane triacrylate (TMPTMA) [159], nanocrystalline cellulose [160], nanofibrous silk fibroin [161], and zwitterionic hydrogels (Z-gels) [162]. All the biomaterials above show potential for use in 3D-printed tissue engineering builds, and the methods of adapting and implementing their use in the medical field are still under development.

With the development of new 3D printing techniques, the medical realm has become more and more interested in future possibilities due to all the advantages that could arise from 3D printing different medical parts. From patient-specific builds to mechanical builds with enhanced bioactivity, 3D-printed biocompatible and functional materials exhibit great advantages and potential. Some materials are already in use in the fields of neurosurgery, orthopedics, tissue engineering, dentistry, bone tissue engineering, prostheses, cardiac surgery, and others.

Moreover, the perspective of VPP’s future potential lies in the recently registered patents. Lymphoid organs and organoids have been designed, 3D printed using polymerization-based 3D printing techniques, and used for immunological tests, functioning as a human-like immune system [163]. Also, patches made from hydrogels have been developed to promote tissue regeneration and healing for dynamic organs such as the lungs, heart, stomach, and bladder. Hydrogels like GelMA and PEGDA are great candidates for this type of build and are also highly compatible with VPP 3D printers [164]. The specificity and precision of these builds can only be obtained by 3D printing, and VPP is one of the most promising techniques in the field that can be applied to these types of biomaterials.

VPP already offers significant advantages compared to other 3D printing techniques due to its high printing speed and accuracy, but its current applications in the medical field are limited by the selection of existing materials. As shown here, adapting existing biomaterials for VPP is a viable method of extending the available list of compatible materials, though challenges still remain. Developing new resins that minimize toxicity by ensuring the efficient removal of unreacted monomers will open up the field to even more applications.

## Figures and Tables

**Table 1:** Properties and medical applications of biocompatible materials used in DLP or SLA 3D printing.

Biocompatible Material	Commercial Name	Additives	Compatible 3D Printer	Physical Properties	Medical Applications	Reference
			Inorganic Biomaterials			
Hydroxyapatite (HAp)	HAp (P100, Baiameng, China)—different mass fraction—50%; 60%; 70%;	Photosensitive resin + dispersant (BYK-2155) + photoinitiator (diphenyl (2,4,6-trimethylbenzoyl phosphine oxide)	Admaflec 130 plus, Admatec, Alkmaar, The Netherlands	- Dependent on the mass fraction (%) 50–70 and the sintering temperature (°C) 1050–1250;	Bone scaffolds, bone grafts, tissue engineering	[49]
	HA/TCP (6:4 ratio; Dentium^®^, Suwon, Korea	Dispersant + acrylic monomer + photo-initiator (Phenylbis phospine oxide)	Cubicon Lux, Cubicon^®^, Sungnam, Korea	- Compressive strengths (MPa): for cubic pores = 6.20 ± 0.8142 MPa; for diamond pores = 2.80–3.60;	Bone scaffolds, bone grafts, tissue engineering	[50]
	HA, Nanjing Duly Biotech Co., Ltd., Nanjing, China	1,6-hexanediol diacrylate + trimethylolpropane triacrylate + 2,4,6-trimethyl-benzoyl + phosphine oxide + Solsperse KOS163	AutoCera, Beijing 10dim Tech. Co., Ltd., Beijing, China	- Solid loading (vol%) = 30; - Relative density (%) = 40%; - Porosity (%) = 60 (55–75); - Shrinkage after print (%): X/Y direction = 29.30 ± 0.07–30.04 ± 0.23; Z direction = 33.3 ± 0.76–32.65 ± 0.86; - Compressive strengths (MPa) = 0.42 ± 0.06–3.32 ± 0.32 (the last 2 properties depend on the shape)—BCC/FCC/TPMS;	Bone scaffolds, bone grafts, tissue engineering	[51]
	LithaBone HA400	Acrylates + methacrylates + radical photoinitiator absorbing in the blue visible region	CeraFab 7500 (Lithoz GmbH, Vienna, Austria)	- Solid loading (vol%) = 46; - Dynamic viscosity (Pa-s at 50s^−1^) = 5–10; - Theoretical density (g/cm^3^) = 3.16; - Relative density (%) = 85;	Variable scaffold geometries for osteogenic applications	[53]
Zirconia	TZ-3Y, Tosho, Japan	Acrylic resin-based IBA (isobornyl acrylate)/HDDA/PNGDA + photoinitator + dispersant + silane coupling agent	Octave Light R1, Octave Light Ltd., Shatin, Hongkong)	- Average linear shrinkage (%): 18–25; - Relative density (%) ≈ 90; - Hardness (HV): 1666–1848; - Flexural Strength (MPa): 368–433;	Not mentioned	[64]
	3YSZ/8YSZ	Acrylic monomer (HDDA/TMPTA/IBOA/HEA/HEMA/PHEA/IDA) + surfactant and photoinitiator of 1 wt.%, all mixed in a polypropylene bottle	Ember (Autodesk, SAN Rafaek, CA, USA)	- 3YSZ: - Refractive index: 2.2; - Tapped density (g/cm^3^): 1.8; - BET (m^2^/g): 6.5; - Relative density (%): above 90; - Average linear shrinkage (%): 29; - 8YSZ: - Refractive index: 2.2; - Tapped density (g/cm^3^): 1.3; - BET (m^2^/g): 12;	Scaffolds with rheological and curing behavior	[65]
	YSZ, TZ-3Y-E grade granules, Tosoh Corporation, Tokyo, Japan	Polyacrylate-based photocurable resin + photoinitiator	IMC-96, Carima Co., Ltd., Daejeon, Korea	- Viscosity (Pa·s): 1.8; - Mechanical strength (MPa): 50.6 ± 6;	Dental implants	[66]
	CY3Z, Saint-Gobain ZirPro, Le Pontet, France	Photocurable resin (ADMATEC Europe BV, Nobelstraat, The Netherlands) + dispersant (Disperbyk-103, BYK Chemie, Wesel, Germany)	ADMAFLEX 130, ADMATEC Europe BV, Nobelstraat, The Netherlands	- Theoretical density (g/cm^3^): 6.05 (for tetragonal zirconia); - Full densification can be obtained by sintering at 1550 °C for 1 h; - Porosity (%): between 38 and 52, dependent on the scaffold needed (the higher the porosity, the lower the compressive strength);	Can be coated with bioactive materials and build bone grafts	[67]
	3 mol % yttria-stabilized zirconia powder (TZ-3YS-E, Tosoh Corp, Tokyo, Japan)	Acrylate monomers + photoinitiators + processing additives; solvent-free	Onestage 6500 (Illuminaid Inc., Seoul, Korea)	- Solid loading (vol.%): 45; - Viscosity (Pa·s): 57 at 30 °C and 13 at 50 °C; - Relative densification (%): 99.02 ± 0.08; - Microhardness (GPa): 12.59 ± 0.47;	High-density zirconia scaffolds	[68]
Lithium disilicate glass–ceramic	VarseoWax CAD/Cast, BEGO, Bremen, Germany	Investing material (Bellavest SH, BEGO, Bremen, Germany)	Varseo, BEGO, Bremen, Germany	Not specified;	Dental crowns	[78]
	Lithium disilicate generic color with 45%Vol solid loading	Not mentioned	CeraFab System S65 Medical (Lithoz, Vienna, Austria)	Not specified;	Dental veneers	[79]
	Formlabs resin, Boston, MA, USA	Not mentioned	Formlabs 2; Formlabs, Boston, MA, USA	Not specified;	Dental veneers	[82]
	VisiJet FTX Green; 3D Systems	Not mentioned	Projet 1200; 3D Systems	Not specified;	Dental veneers	[83]
β-tricalcium phosphate (β-TCP)	LithaBone TCP 380D	Acrylates + methacrylates + radical photoinitiator	CeraFab 7500 (Lithoz GmbH, Vienna, Austria)	- Porosity (vol%): 50–75; - Calcium to phosphorus ratio of stoichiometric TCP is 1.50; - Compressive strengths (MPa): 6.75–44.7; - Relative density (%): 99.5;	Tissue engineering, bone grafts	[89]
	β-TCP (Suzhou Ding’an Technology Co., Ltd., Suzhou, China)	Pyritum + photosensitive resin	3D printing machine developed by Nanjing University of Aeronautics and Astronautics, Nanjing, China	- Mechanical strength (MPa): 5.50 ± 0.24; - Pyritum (wt%): 5; - Porosity (%): 66.11 ± 0.06; - Cumulative degradation rate (%): 8.40; - Microscopic porosity (%); 78.20 ± 0.04; - Minimal cytotoxicity;	Bone grafts/scaffolds for bone defect repair	[90]
	β-TCP from Kunshan Chinese Technology New Material Co., Ltd., Kunshan, China	MgO powder + HDDA + TPGDA + polymeric dispersant 41,100 + P-hydroxyanisole	Autocera-M, Beijing Ten Dimensions Technology Co., Ltd., Beijing, China	- Refractive index: 1.63; - Density (g/cm^3^): 3.07; - β-TCP powder with 0, 1, 3 and 5 wt% MgO; - MgO powders: density 3.58 g/cm^3^, refractive index 1.7; - Theoretical porosity (%): 66.67; - Solid loading (vol%): 50; - Compressive strengths (MPa): 2.84–6.90 accordingly with the quantity of magnesium oxide (0–5 wt%); - Weight loss at 10 weeks (%): 14.20–4.30 accordingly with the quantity of magnesium oxide (0–5 wt%);	Bone grafts, tissue engineering	[92]
Biphasic calcium phosphate	HA + b-TCP 60:40 ratio	Acrylic monomer + dispersant + photocatalyst	Cubicon Lux, Cubicon^®^, Sungnam, Korea	Not specified;	Calvarial scaffolds	[98]
	HA + b-TCP 70:30 Engineering Research Center for Biomaterias of Sichuan University	25% photosensitive resin	AdMaflex 130Plus, AdMatec, The Netherlands	Not specified;	Bioceramic Scaffolds	[102]
	Osteon III block, Genoss, Suwon, Korea	Not mentioned	Cubicon Lux DLP-B12C, Cubicon^®^, Sungnam, Korea	Not specified;	Bone grafts, scaffolds	[103]
	HA + b-TCP 60:40 Kunshan Chinese Technology New Material Co.	HDDA + TPGDA + dispersant + photoinitiator + photoinhibitor (final stage)	Autocera-M, Beijing Ten Dimensions Technology Co., Ltd., Beijing, China	Not specified;	Bioceramic scaffolds	[104]
Organic Biomaterials						
Poly(propylene fumarate) (PPF)	PPF:DEF mixture	Photoinitiators + UV-light absorbing 2-hydroxy-4-methoxybenzophenone	EnvisionTec Microplus Advantage	- Porosity (%): 82; - Viscosity (Pa·s): 1.9; - Mass loss at 30 days (%): 46.0 ± 1.1; - PPF:DEF (wt%) = 50:50; - (PPF:DEF): mixture = 1:1;	Cranial Bone Scaffolds	[108]
	PPF:DEF mixture	Photoinitiators (Irgacure 819 + Irgacure 784) + oxybenzone + additional DEF to bring the final resin composition from 1:3 mass ratio to 1:1 with PPF;	EnvisionTec(Dearborn, MI)	- Intrinsic viscosity at 35 °C (dL/g): 0.0288 ± 0.0009–0.0780 ± 0.0022 (directly proportional with the average molecular mass 0.7 kDa–3.16 kDa); - PPF:DEF = 3:1 mass ratio;	Biocompatible Scaffolds	[111]
	PPF:DEF mixture	Photoinitiators (Irgacure819, Irgacure 784) + HMB + Biogalss	EvisionTec Perfactory 3	- PPF:DEF:Irgacure 819:Irgacure 784 (wt%) = 47.95:47.95:3.00:0.40; - Viscosity (Pa·s): 0.2–1.4 directly proportional with the concentration of Bioglass added (0 wt%–10 wt%);	Bioactive scaffolds	[116]
	PPF:DEF mixture	1,2-propylene glycol + cross-linking inhibitor (Hydroquinone) + catalyst (Zint chloride) + 1.5 wt% BAPO (bisacrylphosphrine oxide)	3D Systems, Valencia, CA; Viper si2 stereolithography	- PPF:DEF (wt%) = 60:40;	Bone tissue engineering, biocompatible scaffolds	[117]
Polycaprolactone (PCL) mixes and polycaprolactone (PCL)-based materials	PCL pellets (Mn ≈ 80,000 g/mol) Sigma-Aldrich	Photoinitiator + photoabsorber (Sudan I) + solvent (dichloromethane)	Self-built 3D DLP printer	- Viscosity (Pa·s): <42; - Low melting temperature (°C): 60; - E_s_ decreases from 4.4 to 0.8 MPa with the increase of C_PCL_;	Self-healing 4D scaffolds	[129]
	Polymerization of ε-caprolactone and diethylene glycol forwarded with the synthesis of polyurethane acrylate	Polyurethane acrylate (PUA) was mixed with two resins (PEGDA and PPG)	Young Optics, Hsinchu City, Taiwan	- ε-caprolactone:DEG = 1:4 molar ratios; - IPDI:2-HEA:PCL = 2:2:2 molar ratio ( for the synthesis of PUA); - PUA1000:PEGDA-PPG = 70:30:0; - Viscosity (Pa·s): <10; - Compressive strength (MPa): 127.66 (for polymerized PUA1000); - Mass loss (%): 30 after 24 h; - Cytotoxicity (%): 63.71% (for PUA1000);	Tissue engineering scaffolds	[122]
	ε-caprolactone monomer (ε-CL, Alfa Aesar, 99%) polymerized	GelMA (The PCL resin did not require use of diluents) + photoinitiator + OrasolYellow dye	Titan 2, Kudo3D, Taiwan	- Molecular weight of PCL (g/mol): 2000; - GelMA:PCL = 70:30 weight ratio-no solvent; - Viscosity (Pa·s): <10 at 30 °C (decreasing with the increasing of the temperature); - Mass loss (%): 42 after 96 h;	Small intestinal villi structure	[124]
Poly(methyl methacrylate) (PMMA)	PMMA (Sigma-Aldrich Co. LLC, Darmstadt, Germany)	MMA (monomer from Fujifilm Wako Pure Chemical Corporation, Osaka, Japan) + EGDMA (cross-linker from Fujifilm, Wako Pure Chemical Corp., Osaka, Japan) + BAPO (photoinitiator from Tokyo Chemical Industry Co., Ltd., Tokyo, Japan)	ELEGOO MARS, ELEGOO INC., Shenzhen, China	- PMMA: EGDMA:MMA ratio (%) = 30:56:14; - Flexural strength (MPa): 84.6 ± 7.1; - Vickers hardness: 21.6 ± 1.9; - Shear bond strength (MPa): 10.5 ± 1.8; - Resin’s degree of conversion (%): 71.5 ± 0.7; - Water sorption (µg/mm^3^): 19.7 ± 0.6; - Solubility (below detection limit); - Cell viability (%): 80.7 ± 6.2 after 10 days;	Dental applications	[134]
	PMMA from NextDent Co. (C&B) for DLP printer, PMMA from Formlabs Co. (Grey Resin, Somerville, MA, USA) for SLA printer	Immersing after print in 100% isopropyl alcohol	DLP (D-150) Nextdent Co. or SLA (Form2) Formlabs Co.	- Flexural strength for DLP (N): ≈1250 and for SLA ≈ 1300;	Dental applications	[137]
	PMMA-resin (NextDent^TM^, NextDent, Utrecht, The Netherlands)	After print rinse with deionized water	DLP 3D printer (MiiCraft Ultra 50X, MIICRAFT, Jena, Germany)	- Compressive strengths before implantation (kPa): 9734.9 ± 4.2; - Compressive strengths after two weeks of implantation (kPa): 7428.5 ± 2.8; - No cytotoxic effect;	3D-printed Tracheostomy tube	[138]
Poly(trimethylene carbonate) PTMC	Trimethylene carbonate (TMC) (Foryou Medical Devices Co., Huizhou, China) that was polymerized	[TMP initiator of TMC + tin(II) 2-ethylhexanoate (Sn(Oct)_2_) + methacrylic anhydride + triethylamine] for polymerization + TPO-L photoinitiator + Orasol orange dye	Not mentioned	- TMC:TMP = 295:1; - Targeted molar mass PTMC: 30 kg mol^−1^; - PTMC:tMA macromer in propylene carbonate (wt%) = 40–70; - Porosity (%): 70; - Viscosity (Pa·s): <132.5; - Pore size: 600 µm; - Compression modulus (MPa): 5.2; - Effective molar mass PTMC: 28.9 kg mol^−1^;	Meniscus implants	[139]
	Trimethylene carbonate (TMC) (Foryou Medical Devices, China) that was polymerized	Gelatin porcine skin (type A) + photoinitator + light absorbent	Asiga Max X27, Australia	- Gel strength 300 g Bloom; - Young Modulus for pure PTMC (MPa): 1.29 ± 0.17; - Young Modulus for Gel-g-PTMC_6(gelcontent≈82%)_ (MPa): 4.62 ± 0.84; - Young Modulus for Gel-g-PTMC_32(gelcontent≈98%)_ (MPa): 0.95 ± 0.09; - Viscosity (MPa·s): 0.24–136.7 (for Gel-g-PTMC_6_ and Gel-g-PTMC_32_, respectively);	Tissue engineering scaffolds	[142]

## Data Availability

No new data were created or analyzed in this study. Data sharing is not applicable to this article.
